# Continued improvement in survival of acute myeloid leukemia patients: an application of the loss in expectation of life

**DOI:** 10.1038/bcj.2016.3

**Published:** 2016-02-05

**Authors:** H Bower, T M-L Andersson, M Björkholm, P W Dickman, P C Lambert, Å R Derolf

**Affiliations:** 1 Department of Medical Epidemiology and Biostatistics, Karolinska Institutet, Stockholm, Sweden; 2 Department of Medicine, Division of Hematology, Karolinska University Hospital Solna and Karolinska Institutet, Stockholm, Sweden; 3 Department of Health Sciences, University of Leicester, Leicester, UK

## Abstract

We evaluated temporal trends in survival of Swedish acute myeloid leukemia (AML) patients diagnosed between 1973 and 2011 using relative survival ratios (RSRs) and a measure called the loss in expectation of life (LEL). RSRs increased most for patients <60 years at diagnosis during the first calendar periods, but between 1997–2005 and 2006–2011 the most pronounced increase was for those aged 61–70 years at diagnosis; RSR changed from 0.16 (95% confidence interval (CI): 0.13–0.19) to 0.28 (95% CI: 0.23–0.33), respectively. The LEL for males aged 35 years at diagnosis was 41.0 (95% CI: 40.1–41.8) years in 1975 and 19.5 (95% CI: 16.4–22.5) years in 2011. For males aged 65 years, the corresponding figures were 13.8 (95% CI: 13.7–14.0) and 12.0 (95% CI: 11.3–12.8). Conditional LEL estimates suggested that patients who survive 5 years postdiagnosis have shorter remaining lifespan than the general population. The proportion of expected life lost (PELL) suggested that male 65-year-old patients lost 75% of their life expectancy in 2005 and 66% if they were diagnosed in 2011. Survival continued to increase to 2011, with larger improvements in those aged 61–70 years at diagnosis. The LEL and PELL are intuitive measures that may be useful in communicating survival statistics to patients, clinicians and health-care providers.

## Introduction

Acute myeloid leukemia (AML) is a disease where the incidence increases with age, the median age at diagnosis being approximately 70 years.^1, 2^ Treatment for AML has remained unchanged for many years, consisting mostly of anthracycline- and cytarabine-based chemotherapy.^3^ Complete remission is achieved in 70–80% of adults aged <60 years, but remission rates in older patients are lower^4^ and the relapse rates are higher. This results in very low long-term survival among the elderly, thus age remains an important prognostic factor. In addition, AML is a heterogeneous disease where treatment response and outcome, at least in part, can be predicted by cytogenetic and molecular genetic findings at diagnosis.^3^ Response to treatment, including the presence of minimal residual disease, is another important prognostic factor.^2, 5, 6, 7, 8^

Most survival data arrive from clinical trials, which are associated with various degrees of patient selection; in particular, elderly and frail patients may not be represented.^9^ Population-based survival data add important information to that obtained from clinical trials. It has been clearly demonstrated from population-based registry data that the results of treatment have improved greatly over the years, especially among younger patients.^10, 11^ Relative survival is now an established measure that is highly useful when comparing cancer survival over time or between groups. An alternative measure is the loss in expectation of life (LEL), which assesses the effect of a diagnosis of cancer on patients' life expectancy and presents survival in terms of the average number of life years lost owing to a diagnosis of cancer.

In recent years, risk stratification of elderly AML patients has improved, and there has been progress in supportive care, including developments in anti-infective prophylaxis and treatment and improved diagnostic methods for opportunistic infections. National guidelines were also launched in Sweden in 2005 to ensure more uniform treatments for AML patients across the country. With these factors in mind, we now extend the follow-up of our previous cohort of 1973–2005 until 2011 with the aim to assess whether survival also improved in recent years and in which age groups. We additionally aimed to determine how the life expectancy of AML patients has changed over time by using LEL.

## Materials and methods

### Cancer registries and patients

The study included AML patients recorded within the nationwide Swedish Cancer Registry established in 1958;^12^ by law, every new incident case of cancer must be reported to this registry. Date of death was obtained by linkage to the Register of Causes of Death. Patients who were diagnosed with AML (ICD7: 2050, 2059, 2060, 2069) between 1 January 1973 and 31 December 2011 were included within the cohort and were followed until their date of death, date of emigration or to the end of follow-up (31 December 2012), whichever occurred first. Only the first diagnosis of AML was considered. Incidental autopsy findings were excluded. Using Systematized Nomenclature of Medicine–Clinical Terms codes (introduced in 1993), we were able to define patients with acute promyelocytic leukemia (APL) between 1993 and 2011. Allogeneic stem cell transplants (allo-SCTs) and autologous stem cell transplants performed in Sweden are reported to the European Society for Blood and Marrow Transplantation (EBMT) register, which was established in 1974. The number and type of SCTs of AML patients diagnosed in Sweden were retrieved from the EBMT register but could not be linked to the individual patient data.

The study was approved by the Stockholm Regional Ethics Review Board. Informed consent was waived, because we had no contact with study patients.

### Statistical methods

Relative survival ratios (RSRs) and LEL were used for quantifying survival of the AML patients. RSR is defined as the observed all-cause survival within the cancer patients under study divided by the expected survival of a comparable group from the general population.^13, 14^ Expected survival for the RSRs was estimated using the Ederer II^15^ method from Swedish population life tables stratified by age, sex and calendar year. The expectation of life is a summary measure that is interpreted as the average number of life years remaining from a certain point in time. The LEL is calculated by subtracting the life expectancy of a cancer patient from the life expectancy of a matched subset of the general population.^16, 17^ This is interpreted as the average number of life years lost owing to a diagnosis of cancer and can provide insight into how a diagnosis of cancer has affected patients' life expectancy. The LEL can also be presented as a proportion of expected life lost (PELL); presenting the LEL as a proportion can be advantageous when making comparisons between groups where the life expectancy in the general population varies. For example, 80-year old patients will have a lower LEL than 35-year-old patients simply because 35-year-olds have a much higher life expectancy and thus more potential life years to lose. It is also of interest to express the LEL conditional on surviving a certain amount of time after diagnosis as excess mortality decreases as survival time increases. The 1- and 5-year conditional LEL measures the expected LEL for patients who are still alive 1 or 5 years postdiagnosis. We present the LEL, 1- and 5-year conditional LEL and PELL to quantify the survival of AML patients. These were estimated from a flexible parametric cure model which included effects of covariates age at diagnosis, year of diagnosis and sex; see Supplementary Materials for a detailed description of modeling methods. All analyses were performed in the Stata Statistical Software, Release 13.1 (StataCorp LP, College Station, TX, USA).

## Results

### Descriptive statistics

A total of 11 598 AML patients (5905 males and 5693 females) were included in the study after excluding incidental autopsy findings (*n*=381) and patients whose first migration date was prior to their diagnosis date (*n*=273). The additional follow-up from 2006 to 2011 contained 2212 AML patients. Analysis was based upon 10 093 total deaths and included 186 patients diagnosed with APL, 74 of whom were diagnosed between 2006 and 2011. Demographic features are displayed in Table 1. Median age at diagnosis for the entire cohort was 69 years and was the same for males and females. There was a continuous increase in median age at diagnosis from 65 years between 1973 and 1980 to 71 years between 2006 and 2011. The numbers of SCTs performed in 1997–2005 and 2006–2011 according to the EBMT register are also presented in Table 1. The largest difference was seen among patients aged 61–70 years, where 10 and 69 allo-SCTs were reported for the two time periods, respectively.

### Temporal trends in relative survival

**Figure 1:**
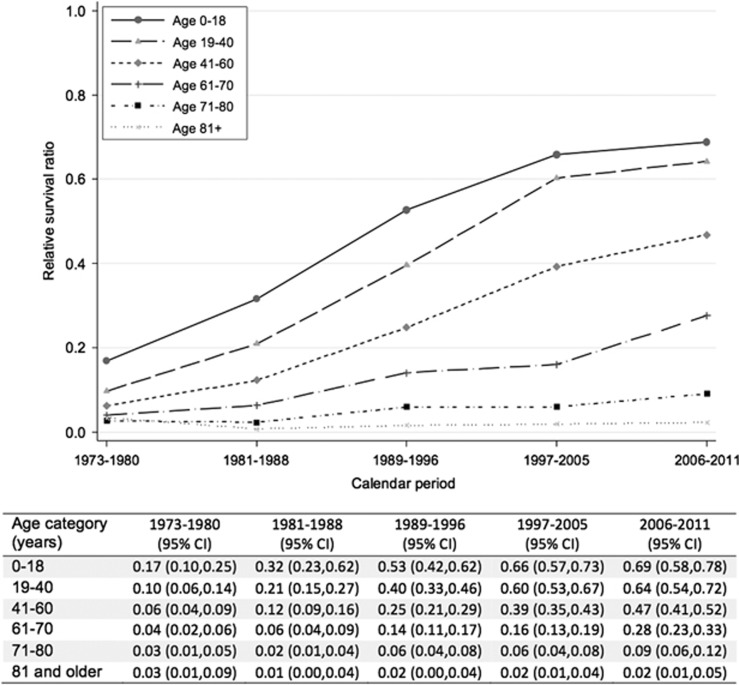
Five-year RSRs of Swedish AML patients stratified by age category over calendar period.

The increase in RSRs for the first four calendar periods as reported previously^10^ continued into the 6 years of additional follow-up for all age groups except the eldest (Figure 1). The increase was most pronounced for patients aged 61–70 years at diagnosis with an increase in RSR from 0.16 (95% confidence interval (CI): 0.13–0.19) to 0.28 (95% CI: 0.23–0.33) between calendar periods 1997–2005 and 2006–2011. An increased relative survival was also observed among patients aged 41–60 years, from 0.39 (95% CI: 0.35–0.43) to 0.47 (95% CI: 0.41–0.52) during the last two calendar periods. The greatest improvement in survival prior to 2005 was seen in those aged ⩽40 years at diagnosis, between 2005 and 2011 the increase in survival for this age group was modest.

### Temporal trends in the life expectancy, LEL and PELL

**Figure 2:**
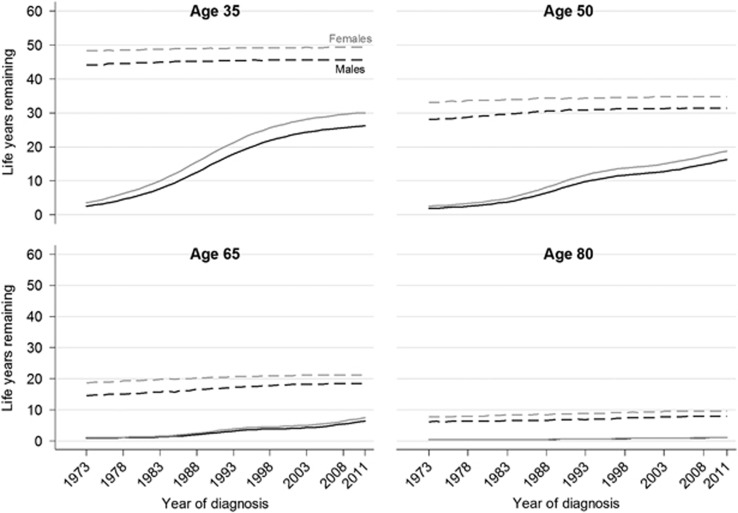
Temporal trends in the life expectancy of the general population (dashed lines) and AML patients (solid lines) in Sweden.

**Figure 3:**
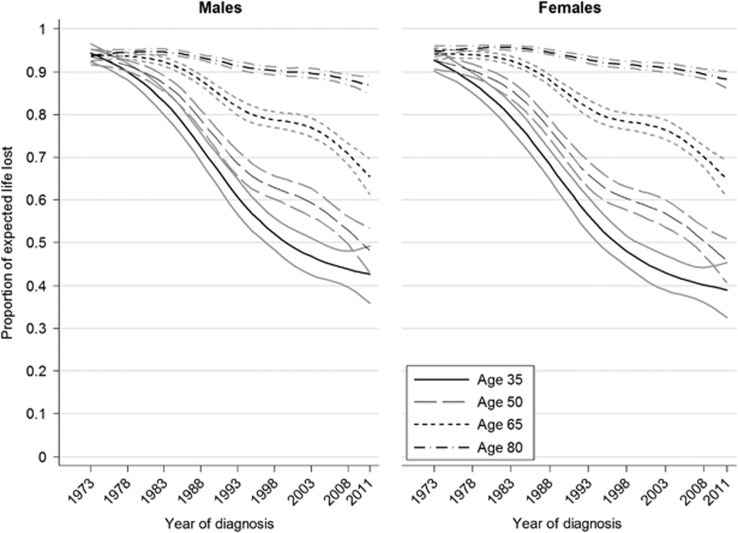
Temporal trends in the PELL of Swedish AML patients, with 95% CIs.

**Figure 4:**
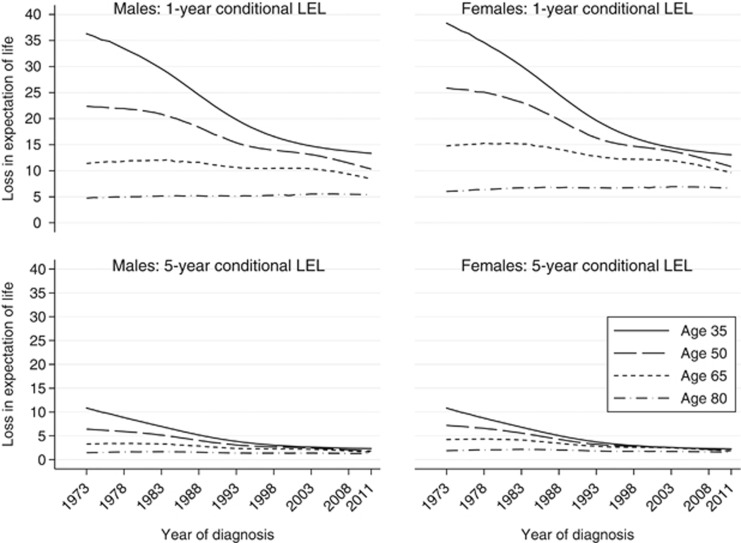
One- and 5-year conditional LEL of Swedish AML patients by sex over year of diagnosis.

The LEL, PELL and conditional LEL are presented for four selected ages: 35, 50, 65, and 80 years (Figures 2, 3, 4). The life expectancy of the general population and that of the AML patients are shown in Figure 2; the difference between these two curves gives the LEL. There was a large increase in the life expectancy of younger AML patients during the study period, while little difference over time was observed for patients aged 80 years. For example, the expected life years remaining of a 35-year-old female AML patient in 1975 and 2011 were 4.5 (95% CI: 3.3–5.6) years and 30.1 (95% CI: 26.9–33.2) years, respectively, whereas the corresponding expected life years remaining for an 80-year-old female AML patient were 0.4 (95% CI: 0.3–0.4) years and 1.1 (95% CI: 0.9–1.3) years. The life expectancy of AML patients, with the exception of those aged 80 years at diagnosis, increased more than that in the general population, which resulted in a continuous decrease in LEL and PELL (Figure 3 and Supplementary Table S1). There was a larger decrease in the LEL for those aged 35 at diagnosis than any of the other ages presented. For example, a diagnosis of AML in a 35-year old male in 1973, on average, shortened the life expectancy by 41.6 (95% CI: 40.7–42.5) years, whereas, for a 35-year old male in 2011, the same diagnosis is predicted to, on average, shorten the life expectancy by 19.5 (95% CI: 16.4–22.5) years. A diagnosis of AML in 1973 and 2011 in male 65-year-old patients would on average reduce the life expectancy by 13.6 (95% CI: 13.4–13.8) and 12.0 (95% CI: 11.3–12.8) years, respectively. The LEL increased marginally over the study period for those patients diagnosed at 80 years of age owing to the fact that the population life expectancy increased more than their AML life expectancy. Results for male and female AML patients were similar; however, the LEL was larger among females. This was due to the difference between life expectancy of the sexes in general being larger than the difference in survival between sexes in AML patients. The PELL indicated that male 65-year-old patients lost 75% of their life expectancy in 2005 and 66% if they were diagnosed in 2011.

The 1- and 5-year conditional LEL were greater for younger patients (Figure 4); similar patterns were seen for males and females. Fewer life years were lost provided patients had survived 5 years postdiagnosis than provided patients had survived 1 year postdiagnosis. For example, a 50-year-old male diagnosed in 2005 who had already survived 1 year postdiagnosis was predicted to have a shortened life expectancy of 12.5 (95% CI: 11.7–13.4) years, whereas, if they had survived 5 years postdiagnosis, they would be predicted to shorten their life expectancy by 2.3 (95% CI: 2.0–2.7) years owing to their diagnosis of AML. A clear age difference can be seen for the 1-year conditional LEL over all years of diagnosis; this difference was much smaller for the 5-year conditional LEL, which was particularly clear when considering diagnoses from 2011 (Figure 4). Despite more improvement among younger patients, the fact remains that younger patients lose more life years than older patients although their relative survival is better than the elderly.

## Discussion

In this large population-based study, we extended our previous analysis of AML survival of patients diagnosed during 1973–2005 to also include patients diagnosed during 2006–2011. In addition, we used both RSRs and LEL to illustrate different aspects of survival.

The additional data from 2006 to 2011 showed a continuing increase in RSRs for all patients except those aged ⩾81 years. The largest increase in relative survival was observed in patients aged 61–70 years, whereas less improvement was observed in younger patients. This pattern was also seen in the PELL estimates with a large improvement in those diagnosed at 65 years between 2006 and 2011. Interestingly, the proportion of patients aged 61–70 years receiving intensive induction chemotherapy has been stable, just >80% between 1997 and 2009 according to data from the Swedish Acute Leukemia Registry.^18^ Thus this improvement cannot be explained by more patients being allocated to induction treatment. However, patients in this age group may have been judged eligible for higher doses of chemotherapy (that is, treated according to full-dose protocols rather than dose reductions). Evidently more patients within this age group were allocated to allo-SCT treatments during this period, as demonstrated by data from the EBMT register. This suggests that more patients aged 61–70 years diagnosed in more recent calendar periods may have been deemed healthy enough to tolerate more intensive treatment. This along with the fact that there have been improvements in health in the general population (European Commission 2008) could suggest that higher survival was seen in these patients than previously owing to better general health and lower comorbidities; less comorbidity also results in better survival after conventional chemotherapy.^19^ This increasing trend in intensive treatment in Sweden may not be possible in other countries with worse health and life expectancy.^20^ In fact, AML survival in Sweden seems to be better than in the United Kingdom^21^ and United States,^10, 11^ where older patients are intensively treated to a lesser extent. Other potential explanations for the improved survival in these patients are better risk stratification of the elderly patients and progress in supportive care in more recent years, including development of better anti-infectious treatment and improved diagnostic methods. National guidelines were also launched for the first time in Sweden in 2005 with the aim to ensure a more uniform treatment over the country.

Although our results indicate that the life expectancy has gradually increased over the study period for patients aged ⩽65 years at diagnosis, there was very little increase in the life expectancy of those AML patients diagnosed at 80 years of age. Intensive treatment is rarely used in AML patients of this age, with supportive care being the common treatment. This suggests that, although improvements in supportive care may have resulted in positive outcomes in some of the AML patient population, it does not necessarily benefit all AML patients.

The LEL decreased for all AML patients in Sweden between 1973 and 2011 and decreased the most for those aged 35 and 50 years at diagnosis. The PELL suggested a greater improvement in patient survival for those aged 50 and 65 years at diagnosis between 2003 and 2011. Although young patients have better survival after diagnosis than the elderly, the LEL and PELL clearly show that younger patients, on average, lose more life years. The LEL and PELL present the average life lost for the whole population under study and may not be optimal measures to illustrate AML survival alone, where patients typically either die within a few years from diagnosis or survive for a long time. Therefore we also present 1- and 5-year conditional LEL, which measure the LEL for patients conditional on the fact that they have survived for 1 or 5 years since diagnosis. Conditional LEL decreased over time for all ages presented apart from those aged 80 years at diagnosis. Conditional LEL estimates toward the end of the study period suggested that patients who survive 5 years post-diagnosis lose approximately the same number of expected life years owing to AML regardless of their age at diagnosis.

Interestingly, young patients surviving 5 years after diagnosis still have their lives shortened by about 2 years. This is most probably explained by treatment-related long-term morbidity and late relapses. The fact that 5-year conditional LEL has decreased substantially for younger patients during the study period suggests there are less complications of applied treatment and fewer relapses. The LEL and PELL are only presented for patients aged ⩾35 years. To estimate these measures in younger patients, further model extrapolation would be required as these patients will have more potential life to lose. This, along with the fact that there are fewer patients diagnosed at a young age, can make estimates from extrapolation in this subset of patients unreliable.

The two measures, RSR and LEL, provide different interpretations of survival from cancer and together contribute to a greater understanding of the impact of a cancer diagnosis. The RSR provides a survival measure of the impact of a diagnosis at a certain time after diagnosis, whereas the LEL provides a measure of the impact a diagnosis has on patients' life expectancy. The least common measure used in practice is the LEL. Although relative survival is highly useful for comparisons of survival, we also believe that using LEL and comparing survival in terms of years is more intuitive and possibly useful for communicating survival statistics. The LEL is calculated by assuming that the life expectancies of patients and the general population are either known or can be estimated.^22^ Patients were followed until the end of 2012, meaning all deaths could not be observed. As a result, model extrapolation was required for calculation of the LEL. We extrapolated using the assumption of cure at 10 years post-diagnosis, that is, the mortality in the cancer population was the same as that in the general population after a certain time point. A plot of the cumulative relative survival suggested cure was as reasonable as the curve plateaued at approximately 9 years. Yu *et al.*^23^ concluded that, provided cure is reasonable, flexible parametric cure models are as good as, if not better, than other modeling methods. Late relapses do occur but are extremely rare with little or no impact.^24^

A major strength of this study is that population-based data were used. The majority of survival studies of patients diagnosed with AML are from clinical trials which often use cohorts that are not representative of the general population;^9^ here we include virtually all AML cases in Sweden diagnosed between 1973 and 2011. The Swedish Cancer Registry has high completeness; in 1998 it was estimated to capture 96% of all cancers in Sweden.^25^ Additionally, unlike previous studies considering patients diagnosed with AML in Sweden,^10, 26^ we chose to model age continuously which makes temporal trends clearer and also reduces the risk of concealing differences within age categories. In contrast, one weakness to using AML diagnoses from the Swedish Cancer Register is that analyses are implemented without considering treatment, comorbidity and other important prognostic factors as this information is often limited within registry-based data.

We are aware that including patients with APL within the cohort may be problematic as, although early death rate for APL patients is high, they generally have a better outcome.^2, 27^ However, the incidence of APL is approximately 3.2% of all AML cases in Sweden,^27^ so our findings are not likely to be affected.

Clearly, an increasing number of patients are cured from AML. Many of them are young and have many years to live. The long-term effect of AML treatment is not well studied, but at least among allografted patients there is a substantial risk of long-term side effects,^28, 29, 30^ including reduced fertility.^31^ It is important to follow-up surviving patients to clarify to what extent there are long-term effects of disease and treatment in order to prevent late side effects.^32^

There is an on-going debate regarding the advantage of intensive treatment among elderly AML patients.^10, 33^ Our data support the notion that patients aged 61–70 years benefit from intensive treatment. Also in patients aged 71–80 years, there is a small group of patients who experience long-term survival or potentially cure, although at a lower level than observed among younger patients,^34, 35^ this would not be possible without intensive induction therapy.

On the other hand, the increase in RSR observed for the last calendar period was small among younger patients. The same proportions of younger patients underwent allo-SCT during the last two calendar periods of the study. In this age group, it is clear that new treatment strategies are needed in order to increase survival.^10, 11^

In summary, we observed a continued increase in the survival of all AML patients except the eldest patients. Our results suggest that those aged 61–70 years at diagnosis may have benefited from intensified treatment and survival trends for these patients seem to be approaching those of the youngest ages. We have also presented the LEL as a useful additional measure that quantifies survival equally, as efficiently and more intuitively than relative survival.

## Figures and Tables

**Table 1:** Demographic features and distribution of allogeneic and autologous stem cell transplantation of patients diagnosed with acute myeloid leukemia in Sweden during 1973–2011

	*1973*–*1980*	*1981*–*1988*	*1989*–*1996*	*1997*–*2005*	*2006*–*2011*	*Total*
Total no. of cases	1771	2234	2327	3054	2212	11 598
Male/female, %	52.2/47.8	50.2/49.8	49.4/50.6	50.8/49.2	52.4/47.6	50.9/49.1
						
*Age in years, n (%)*						
0–18	101 (5.7)	95 (4.3)	95 (4.1)	143 (4.7)	92 (4.2)	526 (4.5)
19–40	197 (11.1)	187 (8.4)	200 (8.6)	198 (6.5)	139 (6.3)	921 (7.9)
41–60	382 (21.6)	429 (19.2)	427 (18.4)	566 (18.5)	382 (17.3)	2186 (18.9)
61–70	426 (24.1)	544 (24.4)	506 (21.7)	584 (19.1)	487 (22.0)	2547 (22.0)
70–80	453 (25.6)	689 (30.8)	712 (30.6)	913 (29.9)	642 (29.0)	3409 (29.4)
⩾81	212 (12.0)	290 (13.0)	387 (16.6)	650 (21.3)	470 (21.3)	2009 (17.3)
						
Median age at diagnosis in years	65	68	69	71	71	69
						
*Allo-SCT/auto-SCT, n*^a^	—	—	—	374/101	377/2	751/103
0–18 years	—	—	—	69/6	45/0	114/6
19–40 years	—	—	—	122/20	98/0	220/20
41–60 years	—	—	—	173/68	165/2	338/70
61–70 years	—	—	—	10/7	69/0	79/7
⩾71 years	—	—	—	0/0	0/0	0/0

Abbreviations: allo-SCT, allogeneic stem cell transplantation; auto-SCT, autologous stem cell transplantation.

a Data on allo-SCT and auto-SCT from the European Society for Blood and Marrow Transplantation register were only obtained for the last two calendar periods.
