# Supplementary material for: Continued improvement in survival of acute myeloid leukemia patients: an application of the loss in expectation of life
Source: Blood Cancer J. 2016 Feb 5;6(2):e390–. doi: 10.1038/bcj.2016.3 (PMC4771966; doi:10.1038/bcj.2016.3)
Supplement: Supplementary Information [file bcj20163x1.docx]

**Methods**

*Detailed Modelling Methods*

The estimates of LEL were obtained from flexible parametric cure models modelling the baseline excess mortality using 7 degrees of freedom. Knots were placed at 0.0001, 0.3, 0.7, 1.5, 4.1, 8.0 and 10.0 years post-diagnosis. Cure was assumed at 10 years post-diagnosis, i.e., the excess hazard was assumed to be zero after 10 years of follow-up. Age at diagnosis and year of diagnosis were both modelled continuously using restricted cubic splines with 4 degrees of freedom and were allowed to be time dependent; sex was additionally included in the model. Interactions between year and age and between sex and age were included within the model. We compared models using the Akaike Information Criteria and the Bayesian Information Criteria. Sensitivity analyses were performed to assess the robustness of results to alternative model choices. Similar results were found when altering the baseline degrees of freedom, the degrees of freedom specified for time-dependent effects, the time of cure between 10 years and 20 years, and the interactions and time-dependent effects specified in the model, see Figures S2, S3 and S4.

**Tables**

**Table S1**: Life expectancy of the general population (years), life expectancy of patients diagnosed with acute myeloid leukaemia (years), loss in expectation of life (LEL) (years) and proportion of expected life lost (PELL), together with 95% confidence intervals, for acute myeloid leukaemia patients diagnosed in Sweden during 1973-2011.

|  | | Age 35 | | Age 50 | | Age 65 | | Age 80 | |
| --- | --- | --- | --- | --- | --- | --- | --- | --- | --- |
|  |  | Males | Females | Males | Females | Males | Females | Males | Females |
| 1975 | Life expectancy | 44.1 | 48.3 | 28.3 | 33.3 | 14.7 | 18.9 | 6.1 | 7.6 |
|  | AML life expectancy | 3.2  (2.3-4.0) | 4.5  (3.3-5.6) | 2.1  (1.6-2.5) | 2.7  (2.2-3.3) | 0.9  (0.8-1.1) | 1.1  (0.9-1.3) | 0.4  (0.3-0.4) | 0.4  (0.3-0.4) |
|  | LEL | 41.0  (40.1-41.8) | 43.8  (42.7-45.0) | 26.3  (25.9-26.7) | 30.6  (30.0-31.2) | 13.8  (13.7-14.0) | 17.8  (17.6-17.9) | 5.7  (5.7-5.8) | 7.3  (7.2-7.3) |
|  | PELL | 0.93  (0.91-0.95) | 0.91  (0.88-0.93) | 0.93  (0.91-0.94) | 0.92  (0.90-0.94) | 0.94  (0.93-0.95) | 0.94  (0.93-0.95) | 0.94  (0.93-0.95) | 0.95  (0.94-0.96) |
|  | 1-year cond. LEL | 35.1  (33.5-36.7) | 36.8  (34.9-38.7) | 22.2  (21.4-23.1) | 25.6  (24.6-26.7) | 11.6  (11.3-12.0) | 15.0  (14.5-15.4) | 4.8  (4.7-4.9) | 6.1  (6.0-6.3) |
|  | 5-year cond. LEL | 10.0  (8.4-11.5) | 9.9  (8.3-11.5) | 6.2  (5.3-7.1) | 7.0  (5.9-8.0) | 3.4  (2.9-3.8) | 4.3  (3.7-4.9) | 1.5  (1.3-1.7) | 1.9  (1.7-2.2) |
| 1985 | Life expectancy | 45.1 | 48.9 | 30.0 | 34.0 | 15.7 | 19.7 | 6.5 | 8.3 |
|  | AML life expectancy | 9.4  (8.0-10.9) | 12.1  (10.4-13.7) | 4.7  (4.1-5.3) | 5.9  (5.2-6.7) | 1.4  (1.3-1.6) | 1.7  (1.5-2.0) | 0.4  (0.3-0.4) | 0.4  (0.3-0.4) |
|  | LEL | 35.7  (34.2-37.1) | 36.8  (35.2-38.5) | 25.3 (24.7-25.9) | 28.1  (27.4-28.9) | 14.3  (14.1-14.4) | 17.9  (17.7-18.1) | 6.2  (6.1-6.2) | 7.9  (7.9-8.0) |
|  | PELL | 0.79  (0.76-0.82) | 0.75  (0.72-0.79) | 0.84 (0.82-0.86) | 0.83  (0.80-0.85) | 0.91  (0.90-0.92) | 0.91  (0.90-0.92) | 0.94  (0.94-0.95) | 0.95  (0.95-0.96) |
|  | 1-year cond. LEL | 27.7 (26.0-29.4) | 28.0 (26.1-29.9) | 20.0  (19.1-20.9) | 22.0  (21.0-23.0) | 11.7  (11.3-12.0) | 14.7  (14.2-15.1) | 5.2  (5.1-5.3) | 6.8  (6.6-6.9) |
|  | 5-year cond. LEL | 6.2  (5.2-7.2) | 6.1  (5.1-7.1) | 4.7  (4.0-5.4) | 5.1  (4.3-5.8) | 3.1  (2.7-3.5) | 3.8  (3.3-4.4) | 1.6  (1.4-1.8) | 2.1  (1.9-2.4) |
| 1995 | Life expectancy | 45.5 | 49.2 | 31.1 | 34.4 | 17.4 | 20.8 | 7.1 | 8.9 |
|  | AML life expectancy | 19.7  (17.8-21.5) | 23.3  (21.3-25.2) | 10.7  (9.8-11.6) | 12.7  (11.7-13.8) | 3.5  (3.1-3.8) | 4.2  (3.8-4.6) | 0.7  (0.6-0.7) | 0.7  (0.6-0.8) |
|  | LEL | 25.8  (24.0-27.7) | 25.9  (24.0-27.8) | 20.4 (19.5-21.3) | 21.7  (20.7-22.8) | 13.9  (13.6-14.3) | 16.6  (16.2-17.0) | 6.4  (6.3-6.5) | 8.2  (8.1-8.2) |
|  | PELL | 0.57  (0.53-0.61) | 0.53  (0.49-0.57) | 0.66  (0.63-0.69) | 0.63  (0.60-0.66) | 0.80  (0.78-0.82) | 0.80  (0.78-0.82) | 0.91  (0.90-0.92) | 0.92  (0.91-0.93) |
|  | 1-year cond. LEL | 18.3  (16.7-19.9) | 18.1  (16.5-19.8) | 14.6  (13.7-15.5) | 15.5  (14.5-16.4) | 10.5  (10.0-10.9) | 12.4  (11.9-13.0) | 5.2  (5.0-5.3) | 6.7  (6.5-6.8) |
|  | 5-year cond. LEL | 3.5  (2.9-4.0) | 3.4  (2.8-3.9) | 2.9  (2.4-3.3) | 3.0  (2.5-3.4) | 2.3  (2.0-2.6) | 2.7  (2.3-3.1) | 1.4  (1.2-1.6) | 1.8  (1.5-2.0) |
| 2005 | Life expectancy | 45.7 | 49.3 | 31.3 | 34.7 | 18.2 | 21.1 | 7.8 | 9.5 |
|  | AML life expectancy | 24.9  (23.1-26.8) | 28.7  (26.8-30.6) | 13.4  (12.5-14.4) | 15.7  (14.7-16.8) | 4.5  (4.2-4.9) | 5.4  (4.9-5.8) | 0.8  (0.8-0.9) | 0.9  (0.8-1.0) |
|  | LEL | 20.8  (18.9-22.6) | 20.5  (18.6-22.4) | 17.9  (17.0-18.9) | 19.0  (17.9-20.0) | 13.7  (13.3-14.1) | 15.7  (15.2-16.2) | 6.9  (6.8-7.0) | 8.6  (8.5-8.6) |
|  | PELL | 0.45  (0.41-0.49) | 0.42  (0.38-0.45) | 0.57  (0.54-0.60) | 0.55  (0.52-0.58) | 0.75  (0.73-0.77) | 0.75  (0.72-0.77) | 0.89  (0.88-0.90) | 0.90  (0.89-0.91) |
|  | 1-year cond. LEL | 14.3  (12.8-15.8) | 14.0  (12.5-15.5) | 12.5  (11.7-13.4) | 13.2  (12.3-14.1) | 10.0  (9.6-10.5) | 11.5  (11.0-12.1) | 5.5  (5.4-5.7) | 6.9  (6.7-7.1) |
|  | 5-year cond. LEL | 2.5  (2.1-3.0) | 2.5  (2.0-2.9) | 2.3  (2.0-2.7) | 2.4  (2.0-2.8) | 2.1  (1.8-2.3) | 2.3  (2.0-2.7) | 1.4  (1.2-1.6) | 1.7  (1.5-1.9) |
| 2011 | Life expectancy | 45.7 | 49.3 | 31.4 | 34.8 | 18.4 | 21.2 | 1.1 | 1.1 |
|  | AML life expectancy | 26.3  (23.2-39.3) | 30.1  (26.9-33.2) | 16.3  (14.6-17.9) | 18.8  (17.0-20.6) | 6.3  (5.6-7.1) | 7.5  (6.5-8.4) | 1.1  (0.9-1.2) | 1.1  (0.9-1.3) |
|  | LEL | 19.5  (16.4-22.5) | 19.2  (16.1-22.4) | 15.2  (13.5-16.8) | 15.9  (14.1-17.7) | 12.0  (11.3-12.8) | 13.7  (12.8-14.7) | 6.9  (6.8-7.1) | 8.5  (8.3-8.6) |
|  | PELL | 0.43  (0.36-0.49) | 0.39  (0.33-0.45) | 0.48  (0.43-0.53) | 0.46  (0.41-0.51) | 0.66  (0.61-0.70) | 0.65  (0.61-0.69) | 0.87  (0.85-0.89) | 0.88  (0.86-0.90) |
|  | 1-year cond. LEL | 13.3  (10.9-15.7) | 13.1  (10.6-15.5) | 10.3  (9.0-11.7) | 10.8  (9.4-12.2) | 8.5  (7.7-9.2) | 9.6  (8.8-10.5) | 5.4  (5.1-5.7) | 6.7  (6.4-7.0) |
|  | 5-year cond. LEL | 2.3  (1.8-2.9) | 2.3  (1.7-2.8) | 1.8  (1.4-2.2) | 1.9  (1.5-2.3) | 1.6  (1.3-1.9) | 1.8  (1.5-2.1) | 1.2  (1.0-1.4) | 1.5  (1.3-1.8) |

**Figures**

**Figure S1:** Temporal trends in the loss in expectation of life, with 95% Cis, for patients diagnosed with acute myeloid leukemia in Sweden between 1973 and 2011.

**Figure S2:** Loss in expectation of life for patients diagnosed at 60 years of age; sensitivity analysis for differing baseline degrees of freedom

**Figure S3**: Loss in expectation of life for patients diagnosed at 60 years of age; sensitivity analysis for differing degrees of freedom for the time-dependent effect

**Figure S3**: Loss in expectation of life for patients diagnosed at 60 years of age; sensitivity analysis for differing points of cure
